# EEG Correlates of Involuntary Cognitions in the Reflexive Imagery Task

**DOI:** 10.3389/fpsyg.2020.00482

**Published:** 2020-03-26

**Authors:** Wei Dou, Allison K. Allen, Hyein Cho, Sabrina Bhangal, Alexander J. Cook, Ezequiel Morsella, Mark W. Geisler

**Affiliations:** ^1^Department of Psychology, University of California, Santa Cruz, Santa Cruz, CA, United States; ^2^Department of Psychology, The Graduate Center, The City University of New York, New York, NY, United States; ^3^Hunter College, The City University of New York, New York, NY, United States; ^4^Department of Psychological Sciences, University of Missouri, Columbia, MO, United States; ^5^Department of Psychology, San Francisco State University, San Francisco, CA, United States; ^6^Department of Neurology, University of California, San Francisco, San Francisco, CA, United States

**Keywords:** involuntary cognition, mental imagery, reflexive imagery task, alpha, inhibition

## Abstract

The Reflexive Imagery Task (RIT) reveals that the activation of sets can result in involuntary cognitions that are triggered by external stimuli. In the basic RIT, subjects are presented with an image of an object (e.g., CAT) and instructed to not think of the name of the object. Involuntary subvocalizations of the name (the RIT effect) arise on roughly 80% of the trials. We conducted an electroencephalography (EEG) study to explore the neural correlates of the RIT effect. Subjects were presented with one object at a time in one condition and two objects simultaneously in another condition. Five regions were defined by electrode sites: frontal (F3–F4), parietal (P3–P4), temporal (T3–T4), right hemisphere (F4–P4), and left hemisphere (F3–P3). We focused on the alpha (8–13 Hz), beta (13–30 Hz), delta (0.01–4 Hz), and theta (4–8 Hz) frequencies.

## Introduction

Upon awakening during the middle of the night, the eyes open and one immediately experiences percepts and urges – the sight of a nightstand, the sound of a clock, and the urge to cover oneself with a blanket. This event, in which *conscious contents*^1^ “just happen” to an observer (Morsella et al., 2016), illustrates what usually occurs in everyday life, when the conscious contents composing the conscious field arise effortlessly, passively, and involuntarily (Morsella et al., 2016). Experiments on perception (e.g., Allen et al., 2016; Firestone and Scholl, 2016) reveal that entry into consciousness of this nature (“involuntary entry,” for short) is influenced by many factors^2^. Urges (e.g., to cover oneself with a blanket), too, can enter consciousness in this involuntary manner (Loewenstein, 1996; Morsella et al., 2009a, b)^3^. Consistent with these observations, several theorists (e.g., Helmholtz, 1856/1961; James, 1890; Freud, 1938; Lashley, 1956; Miller, 1959, 1962; Wegner, 1989) have proposed that, in most circumstances, one is conscious only of what can be regarded as the outputs of mental operations, but not of the operations themselves.

Understanding the mechanisms underlying involuntary entry (Di Lollo et al., 2000; Mathewson et al., 2009) remains one of the most formidable challenges in science (Crick and Koch, 2003). Researchers have begun to investigate one kind of involuntary entry, *stimulus-elicited involuntary entry*, and the factors that influence its occurrence, including stimulus properties (e.g., the salience, novelty, motion, or incentive quality of the stimulus; Gazzaley and D’Esposito, 2007), the subject’s *set* (Bhangal et al., 2018), and the learning history associated with the stimulus (Bhangal et al., 2018). As described in the next section, the Reflexive Imagery Task (RIT; Allen et al., 2013) was developed to investigate such factors.

### Reflexive Imagery Task

The RIT (see review in Bhangal et al., 2016b) focuses on the factors influencing stimulus-elicited involuntary entry, a form of entry that can be time-locked to a stimulus and is experimentally tractable. Stimulus-elicited involuntary entry can be of urges (Morsella et al., 2009a, 2016), percepts (Allen et al., 2016), or even high-level cognitions (Cho et al., 2016). The RIT is based on a rich research tradition, which includes the early experimental approaches of Ach (1905/1951), Eriksen and Eriksen (1974), Stroop (1935), and the work of Wegner (1989) and Gollwitzer (1999). Many aspects of the task stem from “subjective” variants of the Eriksen flanker task (e.g., Morsella et al., 2009a, b; see discussion in Desender et al., 2014; Questienne et al., 2018) in which distractors activate involuntary urges and other conscious contents^4^.

In the initial version of the task (Allen et al., 2013), subjects are instructed to not subvocalize (i.e., say in their head but not aloud) the names of objects (e.g., line drawings from Snodgrass and Vanderwart, 1980) that are presented to them. In Allen et al. (2013), each object was presented one at a time, with each object appearing for 4 s. During the 4 s presentation of the stimulus, subjects could indicate by button press if they happened to subvocalize (involuntarily) the name of the object.

To demonstrate the effect in Allen et al. (2013), we will present momentarily to you, the reader, an object enclosed within parentheses. Your task is to *not* subvocalize (i.e., “say in your head”) the name of the object. Here is the stimulus (▲). When presented with these instructions and then presented by this stimulus, most people cannot suppress the conscious experience of the phonological form of the word “triangle.”

After the presentation of the stimulus, the RIT effect arises after a few moments [*M* = 1,451.27 ms (*SD* = 611.42) in Allen et al., 2013; *M* = 2,323.91 ms (*SD* = 1,183.01) in Cho et al. (2014); *M* = 1,745.97 ms (*SD* = 620.86) in Merrick et al., 2015]. It is important to note that the basic RIT effect, which requires involuntary subvocalization, depends on successful lexical retrieval – a sophisticated, multi-stage process in which only one of tens of thousands of phonological representations is selected for production. The phonological representation, which is based on audition, is selected for production in response to a visual stimulus (e.g., CAT yields/k/,/oe/, and/t/; Levelt, 1989). On the majority of the trials (86% in Allen et al., 2013; 87% in Cho et al., 2014; and 73% in Merrick et al., 2015), subjects fail to suppress such subvocalizations. Subjects report on the majority of trials (∼70%) that the involuntary subvocalization feels “immediate” (Bhangal et al., 2015).

There are more complex versions of the task. In one variant of the RIT (Cho et al., 2016), effects arose even though the involuntary effect involved word-manipulations, as occur in the childhood game of Pig Latin. Subjects were instructed to not transform stimulus words according to a rule (e.g., “CAR” becomes “AR-CAY”). Involuntary transformations (e.g., “SUN” yielding “UN-SAY”) still arose on more than 40% of the trials. In another variant of the RIT (Merrick et al., 2015), subjects were presented with a single object and instructed to (a) not subvocalize the name of the visual object, and (b) not subvocalize the number of letters in the object name. Subjects reported experiencing both kinds of imagery on a considerable proportion of the trials (*M* = 0.30, *SE* = 0.04).

### Behavioral Evidence of the Occurrence of the Imagery

The validity of the RIT effect has been corroborated in several studies. Different kinds of behavioral data support subjects’ reports regarding the occurrence of the mental imagery. For example, in a variant of the basic RIT used in Allen et al. (2013), in which subjects were presented with line drawings and instructed to not subvocalize the name of the drawings, Cushing et al. (2017) instructed subjects to (a) press a button whenever they experience an involuntary subvocalization and (b) press a separate button if the subvocalization (e.g., “cat”) rhymed with a word that subjects had to hold in mind (e.g., “pat”). The accuracy on this rhyme-detection task was high (>80% mean accuracy across trials). This accuracy in performance suggests that subjects did in fact experience phonological imagery (i.e., /k/,/oe/, and/t/) in response to the visual object, for detecting that a word rhymes with another word requires the retrieval of the phonological form of the word, or, at the least, the retrieval of the coda of the word.

A second kind of behavioral evidence stems from Bhangal et al. (2018), a variant of the RIT that was motivated by the research by Ach (1905/1951). Subjects were presented with a visual array of objects and instructed not to count the number of objects presented on the screen. Subjects were instructed to indicate if they still counted the number of objects and to report the sum. In one condition of the experiment, the number of objects was small (2–5 objects). For this condition, when involuntary counting occurred, the counting was very accurate (∼90% mean accuracy). This degree of accuracy suggests that the counting did in fact occur as reported by the subject.

A third kind of behavioral evidence for the occurrence of the imagery stems from a variant of the basic RIT by Bhangal et al. (2015). In this variant of the RIT, some visual objects had names that are high in frequency (e.g., “door”) while others had names that are lower in frequency (e.g., “kite”). The former were more likely to generate involuntary imagery than the latter. Moreover, the RIT effect on a given trial occurred more quickly for high-frequency stimuli than for low-frequency stimuli. Such a frequency effect would be unlikely to arise if the subjects did not experience lexical retrieval.

A fourth kind of behavioral evidence for the occurrence of the imagery stems from a study by Dou et al. (2018). In this study, RIT effects were found to be more likely for some sensory modalities than for others. To take one example, RIT effects were more likely to occur for visual imagery than for verbal imagery, olfactory imagery, or gustatory imagery (Dou et al., 2018). This pattern of results, which is consistent with what is known regarding the generation of imagery across the senses, is unlikely to arise in the absence of imagery.

### Evidence That Subjects Intend to Follow Instructions and That the RIT Effect Is Involuntary

There is evidence that, in the RIT, subjects intend to follow instructions. Specifically, subjects participating in RIT projects often report during debriefing that they (a) intended to follow the instructions (and thereby not have the undesired imagery) and (b) attempted some strategies to try to thwart the RIT effect (Bui et al., in press).

Evidence that the RIT effect is involuntary stems not only from subjects’ reports during debriefing but also from analysis of their trial-by-trial data. First, as mentioned above, the nature of the involuntary subvocalization in the basic version of the RIT is influenced systematically by a stimulus dimension such as word frequency (Bhangal et al., 2015). If this pattern of results were an artifact of experimental demand, it would require for subjects to know subtle ways in which this stimulus dimension should influence the latencies of the behavioral response. Second, in Cho et al. (2014), subjects were instructed to thwart the basic RIT effect by continuously subvocalizing a hum, an activity which could presumably occupy the verbal buffer in working memory and thus interfere with the generation of the involuntary subvocalization elicited by the RIT stimulus. This suppression strategy was unsuccessful, as involuntary subvocalizations still arose on most of the trials (mean proportion ∼0.80). In this variant of the RIT, the intentional activity was the continuous, subvocalized hum, and the unintentional activity was the stimulus-elicited subvocalization. These data revealed that, even when there is sustained imagery that is intentionally generated and unrelated to the RIT stimulus, the RIT effect still arises. In the Cho et al. (2014) study, it would have been difficult for subjects to intentionally subvocalize simultaneously the hum and the name associated with the RIT stimulus (e.g., CAT). In Cho et al. (2014), RIT effects still arose under conditions of cognitive load, in which it is difficult for subjects to implement strategic processing.

One could argue that the RIT effects stems somehow from the nature of the “negative instructions” used in the task, instructions which inform a subject about what he or she is not to do. Such instructions might entice the curiosity of subjects and lead them to voluntarily generate the effect. With this in mind, it is important to point out that the kind of involuntary entry into consciousness found in the RIT arises in tasks that lack any kind of negative instruction to not perform some kind of mental operation. For example, involuntary entry of contents into consciousness arises for ambiguous objects. In one experiment with ambiguous objects (Allen et al., 2016), subjects were instructed to hold in mind, for as long as possible, one way of perceiving an ambiguous object (e.g., Necker cube). Importantly, subjects were never told to not think about alternative ways in which the object could be perceived. Involuntary “perceptual reversals,” involving involuntary entry into consciousness of the rivalrous percept for a given object, occurred on around 80% of the trials, with roughly three such reversals per 30 s trial. In this study, perceptual reversals occurred despite subjects’ intention to perceive only one orientation of the stimulus.

In addition, in the study mentioned above by Dou et al. (2018), it was observed that RIT effects were found to be more likely for some sensory modalities (e.g., vision) than for others (e.g., olfaction). This pattern of results, which reflects what is known regarding the nature of imagery across the senses, is unlikely to arise from intentional processes or demand characteristics.

Additional evidence that the RIT effect is involuntary stems from research revealing that the nature of the effect resembles involuntary, reflex-like processes. For example, in Bhangal et al. (2016a), the RIT effect was less likely to arise (i.e., it habituated) after the repeated presentation of a given object (e.g., CAT presented for ten consecutive trials), which suggests that the RIT effect is activated in a reflex-like manner.

We should add that it is unlikely that RIT effects stem from subjects having long, intentional thought sequences such as, “I should not think of the name of the object, which is *X*,” for, on many trials, the effect arises too quickly to be caused by strategic processing (Allen et al., 2013; Cho et al., 2014). Consistent with this observation, in one version of the RIT, subjects reported on the majority of trials (∼70%) that the involuntary subvocalization felt “immediate” (Bhangal et al., 2015).

Last, it is worth adding that, in all theoretical accounts of involuntary cognitions (including the model of ironic processing by Wegner, 1994; see Footnote 4), it is proposed that the effect “just happens” and is not an artifact of intentional, high-level strategic processes. For example, in one account (Ach, 1905/1951; Bhangal et al., 2016b), merely hearing the word “add” in the instruction “Do not add the following numbers” incidentally increases the activation level of the set to add, which thereby yields “four” in response to the stimuli 2 and 2. The set to subtract, which would have yielded “zero” in response to the same stimuli, did not receive such activation. This account is consistent with the tenets of *parallel distributed processing* (Rumelhart et al., 1986). Of import, in all theoretical accounts of the RIT effect, including those involving cross-modal imagery (see discussion in Dou et al., 2018), the nature of the effect is involuntary.

### Neural Correlates of Stimulus-Elicited Involuntary Entry in the RIT

Neuroimaging evidence, stemming from studies that do not involve the RIT, corroborates subjects’ self-report about the occurrence of private, mental events. In these studies, subjects’ reports about the occurrence of certain mental events [e.g., subvocalizations or ironic processing (Footnote 4)] are preceded by activation in brain areas known to be associated with the occurrence of those mental events (Mason et al., 2007; Mitchell et al., 2007; McVay and Kane, 2010; Pasley et al., 2012; Wyland et al., 2003). For example, a subject’s report of verbal imagery would be preceded by activations in language areas of the brain (e.g., the superior temporal sulcus; Pasley et al., 2012).

However, no project to date has examined the neural correlates of the various processes, including stimulus-elicited involuntary entry, associated with the RIT effect. More generally, data are needed regarding the nature of stimulus-elicited entry involving supraliminal (versus subliminal) stimuli.

In our study, the correlation coefficient measure (a measure that is similar to coherence, Guevara and Corsi-Cabrera, 1996; Guevara et al., 2011) was used to investigate the functional connectivity of the brain regions underlying the electrodes from which we recorded. EEG correlation reflects the similarity of waveforms between two signals and a possible functional relation among different regions of brain.

The data from this EEG project shed light on the basic mechanisms that, in everyday life, engender the contents that occupy our conscious minds. Knowledge of these mechanisms is important for many subfields of psychological science, including those of mind-wandering (Smallwood and Schooler, 2006) and psychopathology, in which it is known that involuntary thoughts (e.g., obsessions and in rumination) can be debilitating (Nolen-Hoeksema et al., 2008).

## Materials And Methods

### Subjects

San Francisco State University students (*n* = 25, female = 19, *M*_age_ = 22.09, *SD*_age_ = 1.65) participated for course credit. The involvement of human subjects in our project was approved by the Institutional Review Board at San Francisco State University. Prior to participation in the study, all subjects provided written consent. All subjects reported having normal health, being right-handed, and having no neurological conditions. The sample size (*n* > 6) was based on the effect size [Cohen’s *d* (on raw proportions) = 3.90; Cohen’s *h* (on raw proportions) = 2.17; Cohen’s *d* (on arcsine transformations of the proportion data) = 2.35], *SD* (0.20), and other aspects of a previous RIT (Cho et al., 2018) that, similar to the present project, presented on each trial two RIT stimuli, instead of just one RIT stimulus. To determine the sample size, we used the program *G^∗^Power 3* (Faul et al., 2007). The input parameters were: Cohen’s *d* = 2.35, one sample *t*-test, tails = one, power = 0.95, and α = 0.05. The output parameters were: non-centrality parameter = 4.70, critical *t* = 2.35, and actual power = 0.96.

### Stimuli and Apparatus

**FIGURE 1:**
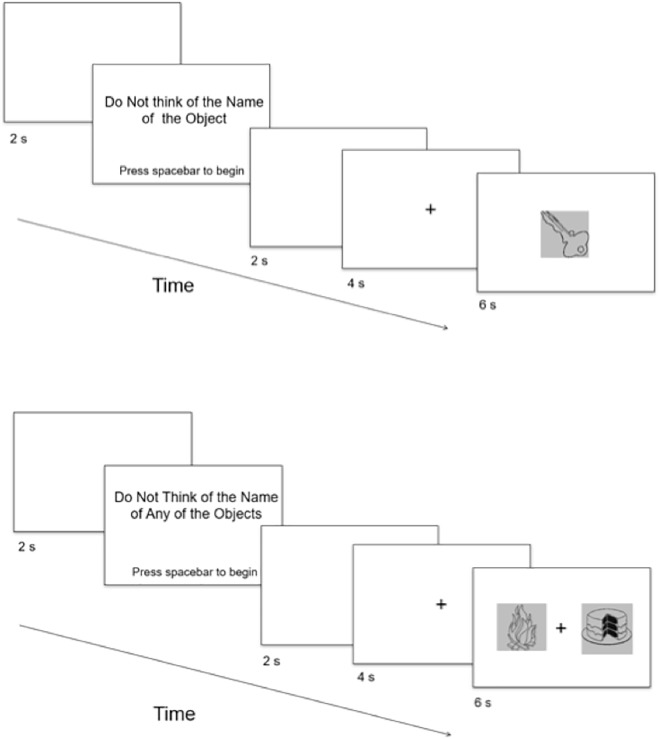
Schematic depiction of trial sequences for the One-Object **(top)** and Two-Object **(bottom)** conditions. Not drawn to scale.

Instructions were presented on a 56 cm monitor using a Dell Optiplex 980 computer with a viewing distance of approximately 60 cm. Stimulus presentation and behavioral data were controlled by SuperLab version 5 (Cedrus Corporation) software. Instructions were presented in black 48-point Helvetica font on a light gray background. In the One-Object block, the stimuli consisted of 37 well-known visual objects (e.g., a key; Figure 1; Appendix) that were displayed at a centered viewing angle of 4.22°× 6.49° (4.42 cm × 6.80 cm). In the Two-Object block, the stimuli consisted of 72 visual objects (e.g., a fire and a cake; Appendix) that were not part of the stimulus set in the One-Object block. On each trial, two visual objects were presented side by side with a fixation-cross (+) between the visual objects (Figure 1). The array of stimuli, which was composed of both visual objects, was presented on the screen with a subtended visual angle of 17.76°× 5.96° (15 cm × 5 cm). Each object occupied the visual angle of 6.56°× 5.96° (5.5 cm × 5 cm). All the stimuli were used successfully in previous research (Snodgrass and Vanderwart, 1980; Morsella and Miozzo, 2002; Allen et al., 2013; Cho et al., 2018).

### Procedures

Subjects were run individually, with the experimenter present, in a sound attenuated and electrically shielded room. The experimenter read all instructions aloud to the subject and verified that the subject understood the instructions before proceeding to the critical trials. Before each block, the subject completed a practice trial that resembled the critical trials. Importantly, the stimuli (HARP, for the One-Object block, and FORK and UMBRELLA, for the Two-Object block) to which the subject responded in the practice trials were not included in any of the critical trials. For the purposes of EEG recording, prior to receiving instructions for the critical trials in each block, the subject completed a baseline trial in which he or she gazed at a fixation-cross presented for 1 min.

In the One-Object block, each subject completed 37 trials in which he or she was instructed to not think of the name of the object that was presented on each trial. The subject was instructed to press the spacebar as soon as possible if he or she happened to think of the name of the object, and to press the spacebar only once per trial. For cases in which the subject did not happen to think of the name of the object, he or she was instructed to do nothing. The subject was informed that the object would remain on the screen for a fixed amount of time, regardless of whether the spacebar was pressed. It was emphasized to the subject that it was important for him or her to keep his or her eyes focused on the center of the screen and to keep his or her fingers rested on the spacebar for the duration of the critical trials. The critical trials commenced as follows (Figure 1). Before each trial, a blank screen (2 s) was presented. Then the subject was presented with the phrase, “*Do Not Think of the Name of the Object*,” in the center of the screen. The subject indicated his or her readiness to begin trials by pressing the spacebar. Trials began with a blank screen for 2 s, followed by a fixation-cross appearing in the center of the screen for 4 s, to prepare the subject for the appearance of the object. Following the fixation-cross, an object appeared for 6 s (according to Cho et al., 2018). Objects were presented individually and in random order, with each object presented only once.

In the Two-Object block, each subject completed 38 trials in which two objects were presented simultaneously on each trial, with one object on the left of the screen and one object on the right of the screen. The subject was instructed to not think of the name of any of the objects that were presented. If he or she did happen to think of the name of any of the objects, then the subject was instructed to indicate by button press each time that he or she happened to think of the name of any of the objects. The subject was told that he or she could indicate by pressing the “z” key on the keyboard if he or she happened to think of the name of the object presented on the left of the fixation-cross, and the character key “/” on the keyboard if he or she happened to think of the name of the object presented on the right of the fixation-cross. The “z” and “/” keys were chosen because (a) they are on opposite sides of a standard keyboard, thereby minimizing the subject’s confusion, and (b) the locations of the keys are equidistant in relation to the spacebar. These two keys were covered by white tape, with “Left” written on the “z” key and “Right” written on the “/” key. Before each trial, the subject was presented with the phrase “*Do Not Think of the Name of Any of the Objects*” in the center of the screen. The subject indicated his or her readiness to begin trials by pressing the spacebar using his or her thumb. Trials began with a blank screen for 2 s, followed by a fixation-cross appearing in the center of the screen for 4 s, to prepare the subject for the appearance of the object. Following the fixation-cross, two objects appeared for 6 s, which was the same duration as in the One-Object block. The subject was instructed to keep his or her eyes focused on the center of the screen where the fixation-cross was presented and to keep his or her index fingers rested on the “z” key and the character key “/” for the duration of the critical trials. Objects were presented in random order, with each object presented only once.

The order of the presentation of the two blocks was fully counterbalanced across all subjects. Once the subject completed the experiment, he or she responded to a series of funneled debriefing questions (following the procedures of Bargh and Chartrand, 2000) designed to help determine whether there was a need to exclude from analysis the data from any subjects. The funneled debriefing included general questions to assess whether (a) the subject was aware of the purpose of the study, (b) the subject had any strategies for completing the task, (c) anything interfered with his or her performance on the task, (d) there were any objects of which the subject did not know the name, (e) the subject often named both objects during trials in which he or she happened to think of the name of either object, (f) the subject ever thought of the name of the object in a language other than English, (g) he or she pressed the spacebar or “z” key and “/” key in such a situation, and (h) he or she had a strategy for completing the task if he or she happened to think of the name of the object in more than one language. From 25 subjects, the data from all subjects were included in the analysis.

### EEG Recording and Analysis

Continuous EEG was recorded from Ag-AgCl electrodes and amplified using the BIOPAC MP150 data acquisition system (BIOPAC Systems, Inc., Goleta, CA, United States). EEG was recorded from eleven electrode sites (Fz, Cz, Pz, F3, F4, C3, C4, T3, T4, P3, and P4) that were positioned in a cap based on the International 10-20 Electrode Placement System and that were referenced to linked mastoids using a forehead iso-ground. Eye artifacts were recorded from two electrodes, with one placed below the right eye and one placed on the outer right ocular canthus. EEG data were collected on a separate (Dell Optiplex GX620) computer using Acknowledge 4.3 software. Data were sampled at 1000 Hz and filtered online with a 0.01–35 Hz bandpass. Impedances were kept below 10 kΩ.

EEG data processing was performed by MATLAB (MathWorks Inc., Natick, MA, United States), EEGLAB (Delorme and Makeig, 2004) and customized MATLAB code. EOG, EMG, and other artifacts were inspected visually and rejected manually. The waveform was bandpass filtered offline by a finite impulse response (FIR) filter and obtained frequency bands for alpha (8–12.9 Hz), beta (13–29.9 Hz), delta (0.1–3.9 Hz), and theta (4–7.9 Hz). For the data of each block, we extracted the first twenty independent 1-s epochs from the baseline period without artifacts. Additional 1-s epochs from the presentation of the fixation-cross in critical trials served as another baseline. Data of critical trials were segmented into 1-s epochs, taken from the first continuous 1-s of data with no artifacts, time-locked to the presentation of the critical stimulus (for the One-Object block) or the stimuli (for the Two-Object block). Only subjects with at least twenty, artifact-free critical trials were included in the analysis. Coherence values were calculated between electrode sites for each region of interest, defined as front (F3 and F4), center (C3 and C4), posterior (P3 and P4), temporal (T3 and T4), left hemisphere (F3 and P3), and right hemisphere (F4 and P4). Coherence values were calculated for each epoch in baseline trials and each epoch of fixation-cross and stimulus/stimuli presentation in the critical trials by obtaining the Spearman correlation coefficients, involving the alpha, beta, delta, and theta waveforms, between the pairs of electrode sites (Godwin et al., 2016). The correlation coefficients were then standardized using the Fisher *r*-to-*z* transformation prior to parametric analysis. This calculation has been shown to be an adequate approximation of coherence in healthy subjects (Guevara and Corsi-Cabrera, 1996).

## Results

### Behavioral Effects: One-Object Condition

The proportion of trials on which subjects had an involuntary subvocalization was 0.77 (*SD* = 0.26, *SE* = 0.05), a proportion that was significantly different from zero, *t*(24) = 14.80, *p* < 0.0001, Cohen’s *h* = 2.14. The same significant result was found with arcsine transformations of the proportion data, *t*(24) = 15.64, *p* < 0.0001. (Arcsine transformations are often used to statistically normalize data that are in the form of proportions). For trials on which there was an RIT effect, the mean latency of this effect was 1,959.55 ms (*SD* = 841.47, *SE* = 168.29).

### Behavioral Effects: Two-Object Condition

The proportion of trials on which subjects had an involuntary subvocalization was 0.76 (*SD* = 0.23, *SE* = 0.05), a proportion that was significantly different from zero, *t*(24) = 16.58, *p* < 0.0001, Cohen’s *h* = 2.14. The same significant result was found with arcsine transformations of the proportion data, *t*(24) = 16.57, *p* < 0.0001. For trials on which there was an RIT effect, the mean latency of this effect was 2,147.92 ms (*SD* = 796.88, *SE* = 159.38).

The RIT effect occurred for both objects on a proportion of 0.40 of the trials (*SD* = 0.39, *SE* = 0.08), which was significantly different from zero, *t*(24) = 5.08, *p* < 0.0001, Cohen’s *h* = 1.37, and was comparable to what was found in Cho et al. (2018), an RIT in which, as in this project, two stimuli were presented on each trial: In that study, involuntary subvocalizations occurred on a high proportion of trials (*M* = 0.78), and the RIT effect arose for both objects on a considerable proportion of the trials (*M* = 0.34). This finding regarding an effect for both objects is also found with arcsine transformations of the proportion data, *t*(24) = 5.92, *p* < 0.0001.

### EEG Results

**FIGURE 2:**
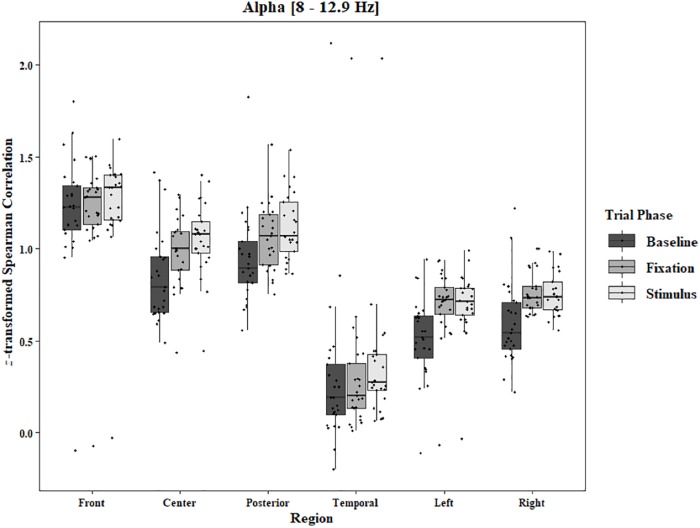
Boxplot showing the median and interquartile ranges of coherence values for front, center, posterior, and temporal regions, and left and right hemipsheres for each phase of the trial in the One-Object Block. Significant increases from baseline to stimulus and baseline to fixation for center (*p* < 0.001 and *p* = 0.002), posterior (*p* < 0.001 and *p* = 0.003), left hemisphere (*p*s < 0.001), and right hemisphere (*p*s < 0.001).

**FIGURE 3:**
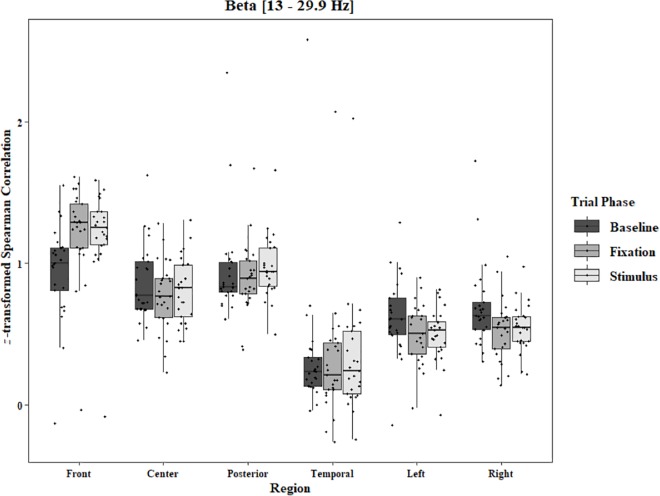
Boxplot showing the median and interquartile ranges of coherence values for front, center, posterior, and temporal regions, and left and right hemipsheres for each phase of the trial in the One-Object Block. Significant increases from baseline to stimulus and baseline to fixation for front region (*p*s < 0.001), and significant decreases in left (*p* = 0.017 and *p* = 0.021) and right (*p* = 0.001 and *p* < 0.001) hemispheres.

**FIGURE 4:**
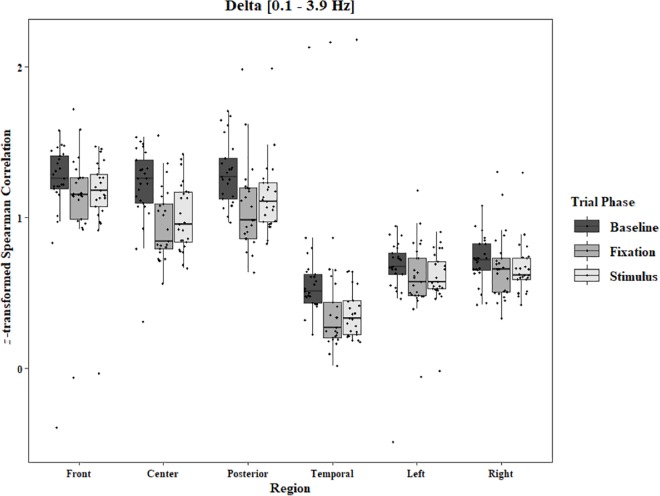
Boxplot showing the median and interquartile ranges of coherence values for front, center, posterior, and temporal regions, and left and right hemipsheres for each phase of the trial in the One-Object Block. Significant decreases from baseline to stimulus and baseline to fixation for center (*p*s < 0.001), posterior (*p*s < 0.001), and temporal (*p*s < 0.001) regions.

**FIGURE 5:**
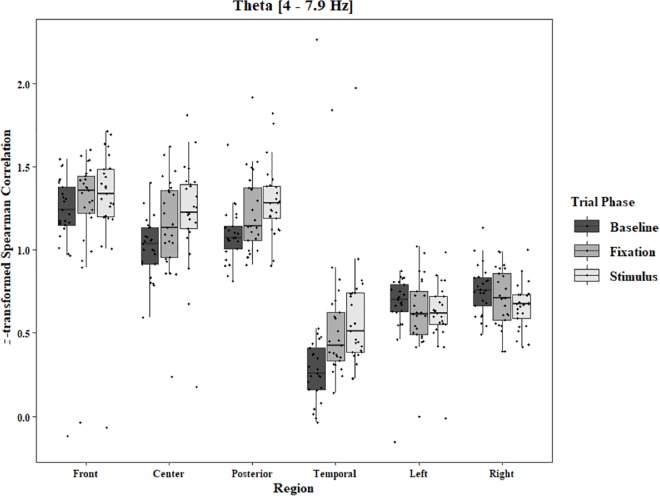
Boxplot showing the median and interquartile ranges of coherence values for front, center, posterior, and temporal regions, and left and right hemipsheres for each phase of the trial in the One-Object Block. Significant increases from baseline to stimulus for front region (*p* = 0.039). Significant increases from baseline to stimlus and baseline to fixation in center (*p* < 0.001 and *p* = 0.005), posterior (*p* < 0.001 and *p* = 0.003), and temporal (*p*s < 0.001) regions.

Coherence data were analyzed with separate repeated-measures ANOVAs for each block and each frequency band on trials in which the subject reported imagery occurring. Levels were Region [front (F3–F4), center (C3–C4), posterior (P3–P4), temporal (T3–T4), left hemisphere (F3–P3), and right hemisphere (F4–P4)] and Trial Phase (baseline, pre-stimulus fixation, and stimulus onset). Reported *F*-values are Greenhouse-Geisser corrected. In the One-Object block, main effects for Region were found in all bands [alpha: *F*(1.25, 30.05) = 44.53, *p* < 0.0001, beta: *F*(1.35, 32.43) = 31.93, *p* < 0.0001, delta: *F*(1.29, 31.05) = 38.46, *p* < 0.0001, theta: *F*(1.34, 32.09) = 49.86, *p* < 0.0001]. Main effects for Trial Phase were found in alpha, delta, and theta [alpha: *F*(1.42, 33.97) = 12.84, *p* = 0.0003, delta: *F*(1.24, 29.76) = 10.87, *p* = 0.001, theta: *F*(1.64, 39.39) = 5.44, *p* = 0.01]. Significant interactions between Region and Trial Phase were found for all bands [alpha: *F*(5.21, 125.04) = 5.07, *p* = 0.0002, beta: *F*(5.48, 131.61) = 15.62, *p* < 0.0001, delta: *F*(4.15, 99.51) = 7.38, *p* < 0.0001, theta: *F*(6.00, 144.03) = 13.89, *p* < 0.0001]. *Post hoc* Tukey tests were performed to examine the differences in coherence data between each region and each trial phase. Increases in alpha coherence from baseline to stimulus onset were found in the center region (*p* < 0.001), the posterior region (*p* < 0.001), the left hemisphere (*p* < 0.001), and the right hemisphere (*p* < 0.001). The same result was found for the comparison between baseline and pre-stimulus fixation for center (*p* = 0.002), posterior (*p* = 0.003), left hemisphere (*p* < 0.001), and right hemisphere (*p* < 0.001; Figure 2). A significant increase in the beta coherence from the baseline to the stimulus onset (*p* < 0.001) and baseline to pre-stimulus fixation (*p* < 0.001) was found in the front region, and significant decreases from baseline to stimulus and baseline to pre-stimulus fixation were found in the left (*p* = 0.017 and *p* = 0.021) and right (*p* = 0.001 and *p* < 0.001) hemispheres (Figure 3). Delta coherence reductions from baseline to stimulus onset and baseline to pre-stimulus fixation were found for the center (*p*s < 0.001), posterior (*p*s < 0.001), and temporal (*p*s < 0.001) regions (Figure 4). Increases of theta coherence, from baseline to stimulus onset, were found for the front (*p* = 0.039), center, posterior, and temporal regions (*p*s < 0.001). Theta increases from baseline to pre-stimulus fixation were found in center (*p* = 0.005), posterior (*p* = 0.003), and temporal (*p* < 0.001) regions (Figure 5). No significant differences were found between pre-stimulus fixation and stimulus onset. See additional results in Supplementary Table S1.

**FIGURE 6:**
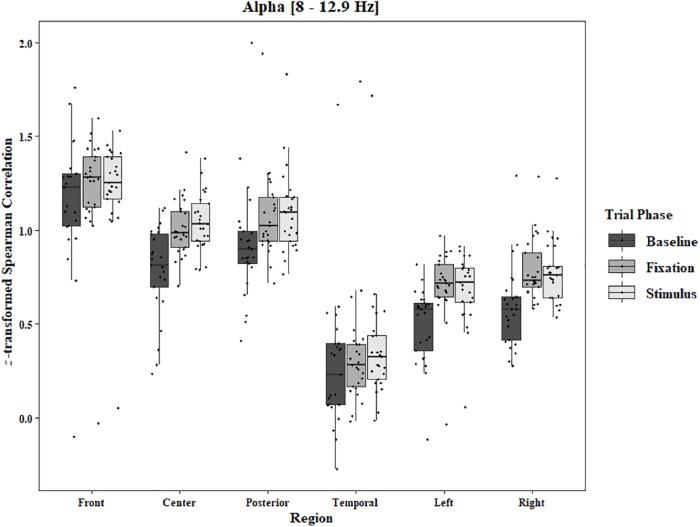
Boxplot showing the median and interquartile ranges of coherence values for front, center, posterior, and temporal regions, and left and right hemipsheres for each phase of the trial in the Two-Object Block. Significant increases from baseline to stimulus and baseline to fixation for center (*p*s < 0.001), posterior (*p*s < 0.001), temporal (*p* < 0.001 and *p* = 0.04), and left hemisphere (*p*s < 0.001), and right hemisphere (*p*s < 0.001).

**FIGURE 7:**
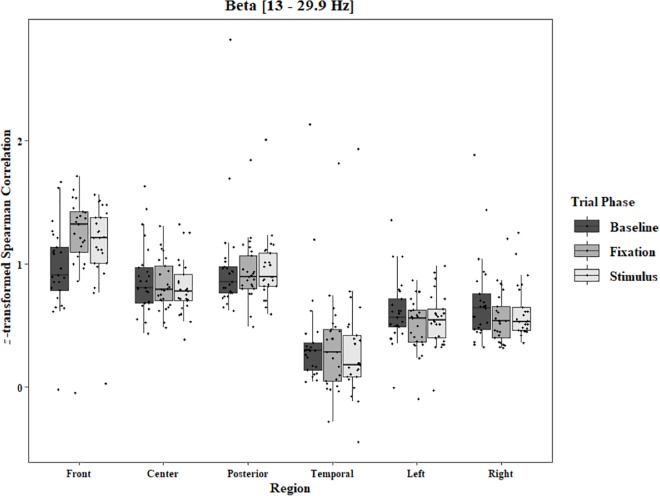
Boxplot showing the median and interquartile ranges of coherence values for front, center, posterior, and temporal regions, and left and right hemipsheres for each phase of the trial in the Two-Object Block. Significant increases from baseline to stimulus and baseline to fixation for front region (*p*s < 0.001). Significant decreases from baseline to fixation in left (*p* = 0.05) and right (*p* = 0.011) hemispheres.

**FIGURE 8:**
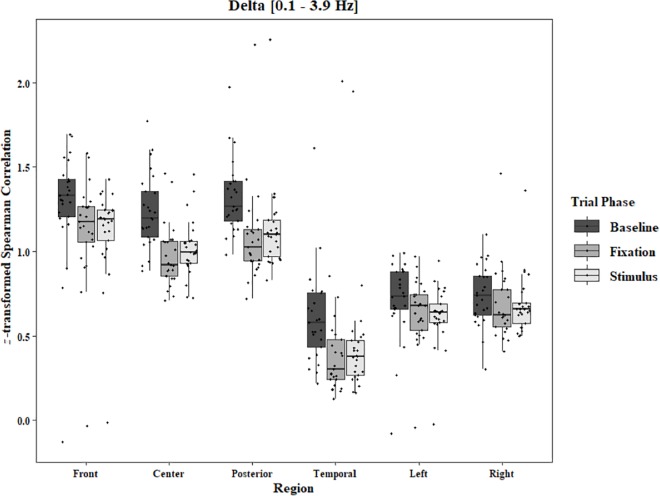
Boxplot showing the median and interquartile ranges of coherence values for front, center, posterior, and temporal regions, and left and right hemipsheres for each phase of the trial in the Two-Object Block. Significant decreases from baseline to stimulus and baseline to fixation for front (*p*s < 0.001), center (*p*s < 0.001), posterior (*p*s < 0.001), and temporal (*p*s < 0.001) regions. Significant decrease from baseline to stimulus for left hemisphere (*p* = 0.045).

**FIGURE 9:**
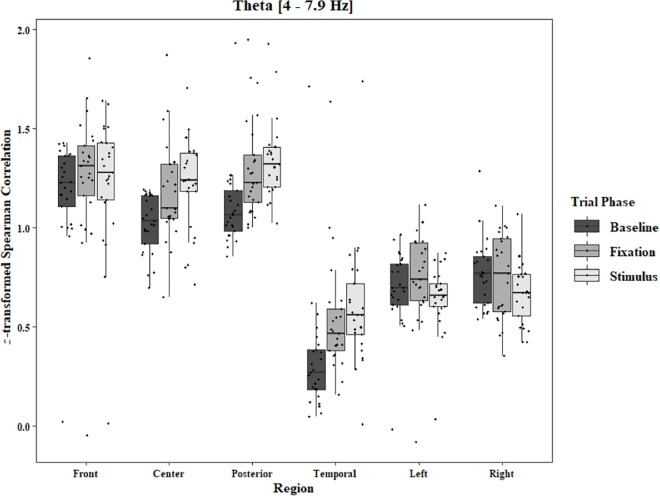
Boxplot showing the median and interquartile ranges of coherence values for front, center, posterior, and temporal regions, and left and right hemipsheres for each phase of the trial in the Two-Object Block. Significant increases from baseline to stimlus and baseline to fixation in center (*p*s < 0.001), posterior (*p*s < 0.001), and temporal (*p*s < 0.001) regions.

In the Two-Object block, main effects for Region were found in all bands [alpha: *F*(1.33, 31.87) = 48.59, *p* < 0.0001, beta: *F*(1.34, 32.05) = 33.39, *p* < 0.0001, delta: *F*(1.28, 30.80) = 44.27, *p* < 0.0001, theta: *F*(1.39, 33.30) = 59.83, *p* < 0.0001]. Main effects for Trial Phase were found in alpha, delta, and theta [alpha: *F*(1.28, 30.81) = 17.94, *p* < 0.0001, delta: *F*(1.24, 29.82) = 17.98, *p* < 0.0001, theta: *F*(1.91, 45.86) = 7.45, *p* = 0.002]. Significant interactions between Region and Trial Phase were found for all bands [alpha: *F*(4.60, 110.29) = 3.79, *p* = 0.004, beta: *F*(4.84, 116.17) = 12.25, *p* < 0.0001, delta: *F*(4.17, 100.16) = 5.90, *p* = 0.0002, theta: *F*(5.57, 133.71) = 11.47, *p* < 0.0001]. *Post hoc* Tukey tests reveal increases in alpha coherence from baseline to stimulus onset in center (*p* < 0.001), posterior (*p* < 0.001), and temporal (*p* = 0.04) regions, and left (*p* < 0.001) and right (*p* < 0.001) hemisphere. Increased coherence from baseline to pre-stimulus fixation was also found in alpha for center, posterior, left hemisphere, and right hemisphere (*p*s < 0.001; Figure 6). Significant rises in beta coherence from baseline to stimulus onset and from baseline to pre-stimulus fixation were observed in the front region (*p*s < 0.001). Decreases in beta from baseline to pre-stimulus fixation were also observed in the right hemisphere (*p* = 0.011) and the left hemisphere (*p* = 0.05; Figure 7). Decreases in delta coherence from baseline to stimulus onset and from baseline to pre-stimulus fixation were found in front, center, posterior, and temporal regions (*p*s < 0.001). A delta decrease from baseline to stimulus onset was observed for the left hemisphere (*p* = 0.045; Figure 8). Increases in theta coherence were found in center (*p*s < 0.001), posterior (*p*s < 0.001), and temporal (*p*s < 0.001) regions from baseline to stimulus onset and from baseline to pre-stimulus fixation (Figure 9). There were no significant differences between pre-stimulus fixation and stimulus onset. See additional results in Supplementary Table S2.

**FIGURE 10:**
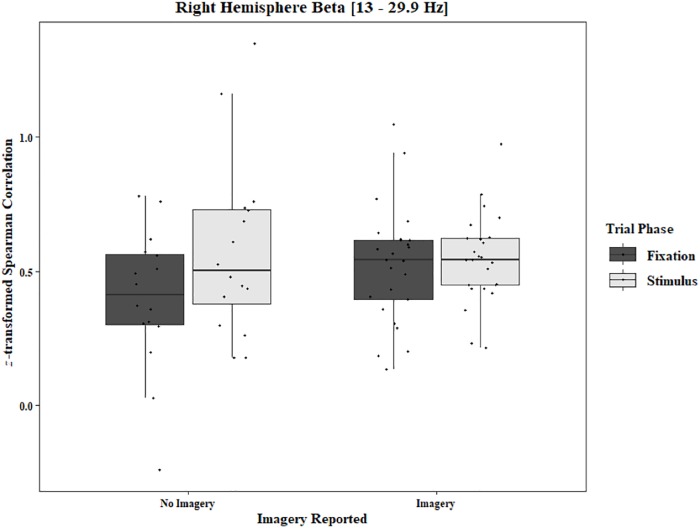
Boxplot showing the median and interquartile ranges of coherence values for trials in which imagery was and was not reported for the phase of each trial in the right hemisphere (One-Object Block). Significant increase from fixation to stimulus for trials in which no imagery was reported (*p* = 0.039), but not when imagery was reported (*p* = 0.98).

**FIGURE 11:**
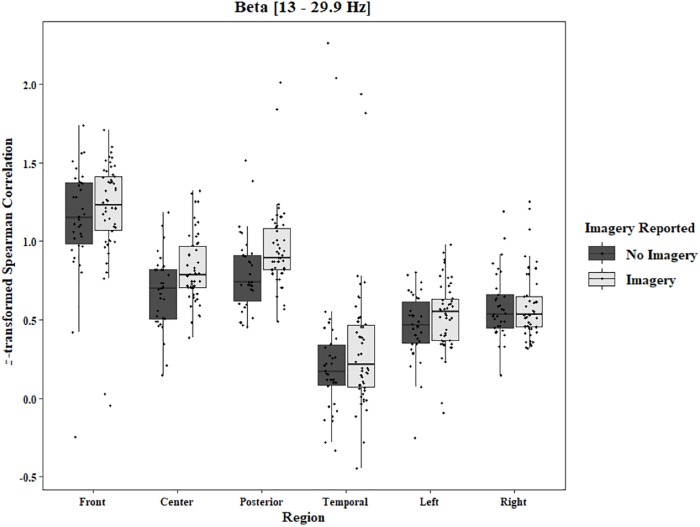
Boxplot showing the median and interquartile ranges of coherence values for front, center, posterior, and temporal regions, and left and right hemipsheres for trials in which imagery was and was not reported in the Two-Object Block. Significantly higher coherence for trials in which imagery was reported for center (*p* = 0.006) and posterior (*p* < 0.001) regions.

To analyze any differences between trials in which imagery was reported and trials in which no imagery was reported, separate three-way ANOVAs for each block and each frequency band were performed using the pre-stimulus fixation for each trial as the baseline. Conditions were Region [front (F3–F4), center (C3–C4), posterior (P3–P4), temporal (T3–T4), left hemisphere (F3–P3), and right hemisphere (F4–P4)], Trial Phase (pre-stimulus fixation and stimulus onset), and Response (Imagery and No Imagery). Significant effects for Region were found for all frequency bands in both the One-Object [alpha: *F*(2.30, 34.47) = 143.62, *p* < 0.0001, beta: *F*(2.82, 42.31) = 121.29, *p* < 0.0001, delta: *F*(2.77, 41.52) = 165.12, *p* < 0.0001, theta: *F*(2.65, 39.81) = 149.76, *p* < 0.0001] and Two-Object [alpha: *F*(1.23, 19.74) = 21.59, *p* < 0.0001, beta: *F*(1.46, 23.40) = 17.89, *p* < 0.0001, delta: *F*(1.19, 19.01) = 15.37, *p* = 0.0006, theta: *F*(1.40, 22.48) = 25.10, *p* < 0.0001] blocks. A significant three-way interaction between Region, Trial Phase, and Response was found for the beta band in the One-Object block, *F*(2.96, 44.44) = 3.49, *p* = 0.02. *Post hoc* Tukey tests reveal the difference to be in the right hemisphere with a significant increase of coherence from pre-stimulus fixation to stimulus onset when no imagery was reported (*p* = 0.039), but no such difference was found in trials in which imagery was reported (*p* = 0.98; Figure 10). For the Two-Object block, a significant effect of Response was found in the beta band, *F*(1, 16) = 5.72, *p* = 0.03, as well as a significant interaction between Region and Response, *F*(3.04, 48.62) = 3.34, *p* = 0.03. *Post hoc* Tukey tests show significantly higher coherence in the center (*p* = 0.006) and posterior regions (*p* < 0.001) for trials in which imagery was reported (Figure 11). No other significant results were found from this analysis.

## Discussion

The present RIT builds incrementally on robust phenomena and on previous versions of the task. Hence, it is the kind of research approach that has been encouraged by leading researchers in the field (e.g., Nosek et al., 2012; Fiedler, 2017). No project to date has examined the neural correlates of the various processes, including stimulus-elicited involuntary entry, associated with the RIT effect. The present data address this gap in the literature and also begin to illuminate a more general process – that involved in the involuntary elicitation of conscious contents through external, supraliminal stimuli. It is important to note that the RIT involves what can be regarded as the Helmholtzian-Freudian unconscious, which operates over, not subliminal stimuli, but supraliminal stimuli (Cushing et al., 2017). It seems that the involuntariness of the conscious contents elicited in the RIT by these supraliminal stimuli reflects, not the exception, but the rule regarding how conscious contents arise. Involuntary entry of contents into consciousness is evident also in binocular rivalry, in earworms (e.g., a tune playing repeatedly in one’s head), and with ambiguous images (e.g., the Necker cube), for which there are involuntary perceptual “reversals” (Allen et al., 2016).

Regarding the new behavioral data, the proportion of trials on which subjects had an involuntary subvocalization was ∼0.76, regardless of whether a stimulus was presented alone (the One-Object condition) or along with another stimulus (the Two-Object condition). The RIT effect occurred for both objects on a proportion of 0.40 of the trials, which replicated the finding of Cho et al. (2018), in which the RIT effect arose for both objects on a considerable proportion of the trials (*M* = 0.34). It is important to note that, unlike in this study, in Cho et al. (2018), there was no One-Object condition. Hence, unlike in the present study, the effects of the two conditions (i.e., One-Object versus Two-Object) could not be compared.

Regarding the EEG data, during the RIT trials, there was increased alpha activity compared to the activity levels found when the eyes were open during the “resting baseline” condition. Alpha is known to be associated with internal thought processes, such as mental imagery (Godwin et al., 2016). It has been proposed that alpha might be associated with top-down inhibitory processes (Cooper et al., 2003; Klimesch et al., 2007). Our results indicate inhibition over central regions and parietal regions, as well as long-range activity in both hemispheres. There was lower baseline alpha coherence when there was no task instruction than during the critical task. There was also increased alpha during the pre-stimulus fixation. Inhibitory processes during the task might also be associated with the increased beta coherence in the frontal region. Beta synchronization in this area has been shown to be an index of response inhibition and cognitive control (Zhang et al., 2008). In the One-Object block, we observed that, in the No-Imagery trials, beta coherence in the right hemisphere increased from pre-stimulus fixation to stimulus onset. In the Two-Object block, beta coherence in the center regions and posterior regions was lower in the No-Imagery trials than in the Imagery trials. These findings are consistent with previous research (Hanslmayr et al., 2012; Waldhauser et al., 2015) revealing that, after the presentation of a cue that instructs subjects to either (a) think about something or (b) not think about something, beta power decreases more in the “No-Think” condition than in the “Think” condition. Regarding the present project, the decreased beta coherence observed during the pre-stimulus fixation might reflect preparation for the forthcoming attempt to suppress subvocalizing. The lower coherence in No-Imagery trials could be related to the successful suppression of subvocalizatons. However, one must be conservative regarding this conclusion because the number of No-Imagery trials is much smaller than the number of Imagery trials.

The significant increases in theta coherence over interhemispheric central, parietal, and temporal regions could be associated with increased efficiency of information processing. It could also be associated with the visual-semantic nature of the images, which are different in many ways from the stimuli (e.g., orthographs) used in English word reading, a skill that activates the left hemisphere (Cheung et al., 2009, 2010). Delta rhythms related to mental imagery and cognitive control have not been studied as thoroughly as have been the other bands. The present results reveal that some cortical decoherence might be related to inhibitory mental processes. Further studies should be conducted to corroborate this initial finding.

### Limitations of the Current Approach

At this stage of understanding, we do not possess a complete understanding of the many aspects associated with the RIT. (See discussion of the component processes of the RIT in Allen et al., 2013). In addition, our approach includes the well-known shortcomings of the measures of introspection and self-report, which are often used in research on consciousness. These measures can be inaccurate as a result of various factors, including (a) subjects basing their reports on a strategy (see discussion in Morsella et al., 2009b), and (b) inaccurate memories of fleeting conscious contents (Block, 2007). Given both the reliability and robustness of the RIT effect (as perhaps experienced by the reader in response to our example involving the triangle), and given corroboratory data (e.g., Bhangal et al., 2015; Cushing et al., 2017), we do not believe that the well-known limitations regarding self-report undermine the validity of our behavioral data.

### The Role of Conscious Imagery in the Mental Simulation of Future Actions

In the theorizing that led to the development of the RIT (e.g., Morsella, 2005; Bargh and Morsella, 2008; Morsella et al., 2016), there is a distinction between the suppressibility of overt behavior and of the generation of conscious contents: One could easily suppress the expression of a given action plan, one cannot so easily suppress the consciously experienced inclinations (e.g., action-related urges) associated with that action plan. For example, one can more easily suppress the act of reaching for someone else’s tasty treat than suppress the desire to have the treat. As Bargh and Morsella (2008) note, inclinations can often be *behaviorally suppressible* but not *mentally suppressible*.

It is clear that external stimuli often activate conscious contents (e.g., percepts, urges, and other inclinations) in a direct, involuntary manner. Often, these conscious contents are insuppressible. So how does adaptive behavior arise from such an arrangement? According to Passive Frame Theory (PFT; Morsella et al., 2016), encapsulated contents can influence behavior collectively only through the conscious field. Without the conscious field, the contents can influence action, but not collectively, yielding instead “un-integrated” actions (Morsella and Bargh, 2011), as sometimes arise when consciousness is decoupled from action in some neurological disorders. When action is in this way decoupled from consciousness, the actions are sophisticated (e.g., manipulating tools in anarchic hand syndrome or in utilization behavior), but they are not influenced by all the kinds of information by which they should be influenced. Conscious contents (e.g., urges, action-related imagery, and other inclinations) that are not selected for action production could be construed as “action options” (Morsella et al., 2016). According to PFT, this arrangement in which such action options are often insuppressible is advantageous, in the course of ontogeny, for instrumental behavior (see discussion in Morsella et al., 2016).

One elaborate form of such action options occurs in the mental imagery involved in mental simulation, which is usually voluntary (see involuntary mental simulation in Cushing et al., 2019). With such imagery (e.g., subvocalization of a funny comment), one can learn about potential action outcomes (e.g., the comment would not be appropriate) without incurring the costs or risks of performing the actions. This is consistent with the view of Thorndike (1905), who concludes, “The function of thoughts and feelings is to influence actions… Thought aims at knowledge, but with the final aim of using the knowledge to guide action” (p. 111).

Together, the present behavioral data and neural data begin to illuminate the mechanisms that, in everyday life, underlie the entry into consciousness of one content versus another content. Knowledge of these basic mechanisms is important for many subfields of psychological science, including those of mind-wandering and psychopathology.

## Data Availability Statement

The datasets for this article are not publicly available because they remain the object of secondary analyses that might lead to new findings and additional, subsequent research reports. Requests to access the datasets should be directed to corresponding author.

## Ethics Statement

The studies involving human subjects were reviewed and approved by The Internal Review Board Committee at San Francisco State University. The subjects provided their written informed consent to participate in this study.

## Author Contributions

WD, AA, HC, SB, AC, EM, and MG developed the concept, helped design and execute the study and contributed to the writing of the manuscript.

## Conflict of Interest

The authors declare that the research was conducted in the absence of any commercial or financial relationships that could be construed as a potential conflict of interest.

## Figures and Tables

**TABLE 1:** Stimuli for one-object block.

Anchor	Flag	Mouse	Sword
Apple	Glasses	Nose	Television
Baseball bat	Goat	Pencil	Tie
Book	Hammer	Pipe	Tower
Bowl	Hand	Radio	Wheel
Candle	Hat	Rake	Whistle
Cat	Iron	Ruler	Wrench
Chair	Key	Saw	
Doll	Ladder	Scissors	
Envelope	Lamp	Snail	
			

**TABLE 2:** Stimuli for two-object block.

Ambulance	Devil	Knife	Snowflake
Angel	Dog	Lightening	Snowman
Axe	Dynamite	Lion	Spider
Ball	Fire	Lips	Star
Balloon	Fireworks	Mosquito	Sun
Bed	Flower	Motorcycle	Swan
Bicycle	Fly	Necklace	Swing
Bird	Gravestones	Noose	Tank
Bomb	Grenade	Paintbrush	Thorn
Bullet	Guillotine	Poison	Tiger
Butterfly	Guitar	Pumpkin	Top
Cake	Gun	Rabbit	Tornado
Candy	Heart	Rainbow	Tree
Cannon	House	Razor	Trophy
Cigarette	Jail	Ring	Volvano
Claw	Jaws	Robber	Wagon
Cockroach	Jewel	Scorpion	Wasp
Coffin	Kite	Shark	Waterfall
Crown	Kitten	Snake	World

## Footnotes

1 Each thing one is conscious of is referred to as a “conscious content” (e.g., a yellow afterimage or nausea). The *conscious field* is composed of all the conscious contents activated at one time.

2 The variables influencing entry of a particular stimulus into consciousness include the salience, novelty, motion, or incentive/emotional quality of the stimulus (Gazzaley and D’Esposito, 2007; Goodhew, 2017). The mechanisms underlying involuntary entry seem to vary across modalities. For instance, a “pop-out” effect (Treisman and Gelade, 1980) may influence entry in vision, but it is less likely to do so in olfaction.

3 Investigations on action control have illuminated that involuntary entry of urges can arise from bodily needs (Loewenstein, 1996) and from the activation of conflicting action plans (Lewin, 1935; Morsella et al., 2009a, b; Desender et al., 2014; Questienne et al., 2018). Moreover, metacognitions (e.g., action-related urges) can enter consciousness insuppressibly as a function of set and the presentation of external stimuli (Garcia et al., 2016).

4 The flanker task precedes research on *ironic processing*, which is associated with failures of self-regulation (e.g., in dieting; Wegner, 1989). Ironic effects arise when one thinks about a given mental representation (e.g., memory or mental imagery) while attempting to not think about that representation (The ironic effect was noted long ago by Dostoevsky, 1863/2008.) It should be mentioned that the RIT, unlike ironic processing, was designed to investigate, not failures in self-regulation, but the nature of involuntary entry from sets and external stimuli. In short, research on the RIT and on ironic processing stem from different theoretical backgrounds and are concerned with different phenomena and with answering different questions. (For reviews of ironic processing and thought suppression, see Wegner, 1989; Rassin, 2005).
