# Supplementary material for: EEG Correlates of Involuntary Cognitions in the Reflexive Imagery Task
Source: Front Psychol. 2020 Mar 26;11:482. doi: 10.3389/fpsyg.2020.00482 (PMC7113402; doi:10.3389/fpsyg.2020.00482)
Supplement: Supplementary file 1 [file Table_1.docx]

One-Object Block: Alpha

| Region | Baseline | Pre-Stimulus Fixation | *t* | *p* |
| --- | --- | --- | --- | --- |
| Center | 0.85 ± 0.05 | 0.98 ± 0.19 | -3.53 | 0.002** |
| Front | 1.20 ± 0.07 | 1.21 ± 0.30 | -0.29 | 0.95 |
| Left | 0.52 ± 0.04 | 0.70 ± 0.20 | -4.73 | <0.001** |
| Right | 0.59 ± 0.05 | 0.76 ± 0.11 | -4.51 | <0.001** |
| Rear | 0.94 ± 0.05 | 1.07 ± 0.18 | -3.33 | 0.003** |
| Temporal | 0.30 ± 0.09 | 0.31 ± 0.40 | -0.43 | 0.90 |

One-Object Block: Alpha

| Region | Baseline | Stimulus | *t* | *p* |
| --- | --- | --- | --- | --- |
| Center | 0.85 ± 0.05 | 1.05 ± 0.20 | -5.37 | <0.001** |
| Front | 1.20 ± 0.07 | 1.24 ± 0.30 | -1.05 | 0.55 |
| Left | 0.52 ± 0.04 | 0.70 ± 0.19 | -4.79 | <0.001** |
| Right | 0.59 ± 0.05 | 0.75 ± 0.11 | -4.17 | <0.001** |
| Rear | 0.94 ± 0.05 | 1.12 ± 0.18 | -3.33 | 0.003** |
| Temporal | 0.30 ± 0.09 | 0.37 ± 0.38 | -1.97 | 0.12 |

One-Object Block: Beta

| Region | Baseline | Pre-Stimulus Fixation | *t* | *p* |
| --- | --- | --- | --- | --- |
| Center | 0.87 ± 0.27 | 0.77 ± 0.25 | 2.20 | 0.07 |
| Front | 0.94 ± 0.34 | 1.22 ± 0.33 | -6.58 | <0.001** |
| Left | 0.62 ± 0.27 | 0.51± 0.21 | 2.70 | 0.02* |
| Right | 0.69 ± 0.30 | 0.52 ± 0.22 | 4.08 | <0.001** |
| Rear | 0.95 ± 0.36 | 0.89 ± 0.25 | 1.26 | 0.42 |
| Temporal | 0.34 ± 0.50 | 0.30 ± 0.44 | 0.99 | 0.58 |

One-Object Block: Beta

| Region | Baseline | Stimulus | *t* | *p* |
| --- | --- | --- | --- | --- |
| Center | 0.87 ± 0.27 | 0.80 ± 0.23 | 1.52 | 0.29 |
| Front | 0.94 ± 0.34 | 1.21 ± 0.31 | -6.51 | <0.001** |
| Left | 0.62 ± 0.27 | 0.50 ± 0.19 | 2.77 | 0.02* |
| Right | 0.69 ± 0.30 | 0.54 ± 0.17 | 3.57 | 0.001** |
| Rear | 0.95 ± 0.36 | 0.97 ± 0.22 | -0.55 | 0.85 |
| Temporal | 0.34 ± 0.50 | 0.33 ± 0.43 | 0.24 | 0.97 |

One-Object Block: Delta

| Region | Baseline | Pre-Stimulus Fixation | *t* | *p* |
| --- | --- | --- | --- | --- |
| Center | 1.21 ± 0.27 | 0.95 ± 0.24 | 6.41 | <0.001** |
| Front | 1.21 ± 0.38 | 1.14 ± 0.32 | 1.78 | 0.18 |
| Left | 0.65 ± 0.27 | 0.61 ± 0.23 | 1.04 | 0.55 |
| Right | 0.71 ± 0.15 | 0.66 ± 0.22 | 1.25 | 0.42 |
| Rear | 1.29 ± 0.22 | 1.07 ± 0.29 | 5.71 | <0.001** |
| Temporal | 0.60 ± 0.35 | 0.40 ± 0.42 | 4.80 | <0.001** |

One-Object Block: Delta

| Region | Baseline | Stimulus | *t* | *p* |
| --- | --- | --- | --- | --- |
| Center | 1.21 ± 0.27 | 1.00 ± 0.22 | 5.21 | <0.001** |
| Front | 1.21 ± 0.38 | 1.15 ± 0.29 | 1.49 | 0.30 |
| Left | 0.65 ± 0.27 | 0.61 ± 0.18 | 1.01 | 0.57 |
| Right | 0.71 ± 0.15 | 0.66 ± 0.18 | 1.44 | 0.32 |
| Rear | 1.29 ± 0.22 | 1.13 ± 0.24 | 4.02 | <0.001** |
| Temporal | 0.60 ± 0.35 | 0.43 ± 0.39 | 4.12 | <0.001** |

One-Object Block: Theta

| Region | Baseline | Pre-Stimulus Fixation | *t* | *p* |
| --- | --- | --- | --- | --- |
| Center | 1.02 ± 0.17 | 1.15 ± 0.30 | -3.21 | 0.005** |
| Front | 1.21 ± 0.32 | 1.27 ± 0.33 | -1.49 | 0.30 |
| Left | 0.67 ± 0.20 | 0.63 ± 0.22 | 1.03 | 0.56 |
| Right | 0.76 ± 0.15 | 0.71 ± 0.17 | 1.16 | 0.48 |
| Rear | 1.09 ± 0.17 | 1.23 ± 0.24 | -3.38 | 0.003** |
| Temporal | 0.34 ± 0.43 | 0.52 ± 0.34 | -4.45 | <0.001** |

One-Object Block: Theta

| Region | Baseline | Stimulus | *t* | *p* |
| --- | --- | --- | --- | --- |
| Center | 1.02 ± 0.17 | 1.22 ± 0.32 | -4.97 | <0.001** |
| Front | 1.21 ± 0.32 | 1.31 ± 0.34 | -2.47 | 0.04* |
| Left | 0.67 ± 0.20 | 0.61 ± 0.19 | 1.32 | 0.38 |
| Right | 0.76 ± 0.15 | 0.66 ± 0.14 | 2.36 | 0.05 |
| Rear | 1.09 ± 0.17 | 1.29 ± 0.21 | -4.90 | <0.001** |
| Temporal | 0.34 ± 0.43 | 0.60 ± 0.35 | -6.28 | <0.001** |

**Table 1.** Transformed Spearman Correlation values for front, center, posterior, and temporal regions, and left and right hemispheres, in four different frequency bands in One-Object Block. Note: **p* < 0.05, ***p* < 0.01.
