# Supplementary material for: EEG Correlates of Involuntary Cognitions in the Reflexive Imagery Task
Source: Front Psychol. 2020 Mar 26;11:482. doi: 10.3389/fpsyg.2020.00482 (PMC7113402; doi:10.3389/fpsyg.2020.00482)
Supplement: Supplementary file 2 [file Table_2.docx]

Two-Object Block: Alpha

| Region | Baseline | Pre-Stimulus Fixation | *t* | *p* |
| --- | --- | --- | --- | --- |
| Center | 0.78 ± 0.24 | 1.01 ± 0.15 | -5.50 | <0.001** |
| Front | 1.16 ± 0.35 | 1.23 ± 0.30 | -1.74 | 0.19 |
| Left | 0.49 ± 0.20 | 0.70 ± 0.19 | -5.18 | <0.001** |
| Right | 0.59 ± 0.22 | 0.79 ± 0.16 | -4.91 | <0.001** |
| Rear | 0.93 ± 0.31 | 1.08 ± 0.24 | -3.75 | <0.001** |
| Temporal | 0.27 ± 0.37 | 0.34 ± 0.35 | -1.70 | 0.21 |

Two-Object Block: Alpha

| Region | Baseline | Stimulus | *t* | *p* |
| --- | --- | --- | --- | --- |
| Center | 0.78 ± 0.24 | 1.04 ± 0.15 | -6.30 | <0.001** |
| Front | 1.16 ± 0.35 | 1.23 ± 0.28 | -1.70 | 0.21 |
| Left | 0.49 ± 0.20 | 0.68 ± 0.18 | -4.70 | <0.001** |
| Right | 0.59 ± 0.22 | 0.77 ± 0.16 | -4.51 | <0.001** |
| Rear | 0.93 ± 0.31 | 1.09 ± 0.22 | -4.15 | <0.001** |
| Temporal | 0.27 ± 0.37 | 0.37 ± 0.33 | -2.43 | 0.04* |

Two-Object Block: Beta

| Region | Baseline | Pre-Stimulus Fixation | *t* | *p* |
| --- | --- | --- | --- | --- |
| Center | 0.88 ± 0.29 | 0.83 ± 0.21 | 1.03 | 0.56 |
| Front | 0.96 ± 0.35 | 1.23 ± 0.34 | -5.98 | <0.001** |
| Left | 0.61 ± 0.27 | 0.50 ± 0.22 | 2.38 | 0.05* |
| Right | 0.69 ± 0.35 | 0.56 ± 0.22 | 2.94 | 0.01** |
| Rear | 0.97 ± 0.44 | 0.94 ± 0.26 | 0.80 | 0.71 |
| Temporal | 0.38 ± 0.44 | 0.33 ± 0.40 | 1.12 | 0.50 |

Two-Object Block: Beta

| Region | Baseline | Stimulus | *t* | *p* |
| --- | --- | --- | --- | --- |
| Center | 0.88 ± 0.29 | 0.81 ± 0.23 | 1.48 | 0.31 |
| Front | 0.96 ± 0.35 | 1.17 ± 0.33 | -4.50 | <0.001** |
| Left | 0.61 ± 0.27 | 0.55 ± 0.22 | 1.39 | 0.35 |
| Right | 0.69 ± 0.35 | 0.61 ± 0.22 | 1.81 | 0.17 |
| Rear | 0.97 ± 0.44 | 0.96 ± 0.28 | 0.30 | 0.95 |
| Temporal | 0.38 ± 0.44 | 0.30 ± 0.44 | 1.73 | 0.20 |

Two-Object Block: Delta

| Region | Baseline | Pre-Stimulus Fixation | *t* | *p* |
| --- | --- | --- | --- | --- |
| Center | 1.24 ± 0.22 | 0.97 ± 0.19 | 6.97 | <0.001** |
| Front | 1.27 ± 0.36 | 1.13 ± 0.31 | 3.76 | <0.001** |
| Left | 0.71 ± 0.24 | 0.64 ± 0.20 | 1.88 | 0.15 |
| Right | 0.74 ± 0.18 | 0.69 ± 0.22 | 1.40 | 0.34 |
| Rear | 1.32 ± 0.22 | 1.08 ± 0.29 | 6.35 | <0.001** |
| Temporal | 0.61 ± 0.28 | 0.42 ± 0.38 | 4.77 | <0.001** |

Two-Object Block: Delta

| Region | Baseline | Stimulus | *t* | *p* |
| --- | --- | --- | --- | --- |
| Center | 1.24 ± 0.22 | 1.02 ± 0.17 | 5.74 | <0.001** |
| Front | 1.27 ± 0.36 | 1.12 ± 0.28 | 4.15 | <0.001** |
| Left | 0.71 ± 0.24 | 0.62 ± 0.18 | 2.42 | 0.04* |
| Right | 0.74 ± 0.18 | 0.69 ± 0.18 | 1.44 | 0.32 |
| Rear | 1.32 ± 0.22 | 1.14 ± 0.27 | 4.91 | <0.001** |
| Temporal | 0.61 ± 0.28 | 0.44 ± 0.35 | 4.37 | <0.001** |

Two-Object Block: Theta

| Region | Baseline | Pre-Stimulus Fixation | *t* | *p* |
| --- | --- | --- | --- | --- |
| Center | 1.02 ± 0.15 | 1.18 ± 0.25 | -3.69 | <0.001* |
| Front | 1.17 ± 0.28 | 1.26 ± 0.35 | -1.89 | 0.15 |
| Left | 0.69 ± 0.19 | 0.75 ± 0.24 | -1.29 | 0.40 |
| Right | 0.77 ± 0.17 | 0.75 ± 0.21 | 0.48 | 0.88 |
| Rear | 1.11 ± 0.21 | 1.30 ± 0.25 | -4.47 | <0.001* |
| Temporal | 0.34 ± 0.32 | 0.54 ± 0.30 | -4.60 | <0.001* |

Two-Object Block: Theta

| Region | Baseline | Stimulus | *t* | *p* |
| --- | --- | --- | --- | --- |
| Center | 1.02 ± 0.15 | 1.22 ± 0.24 | -4.6140421 | <0.001** |
| Front | 1.17 ± 0.28 | 1.23 ± 0.33 | -1.2975833 | 0.40 |
| Left | 0.69 ± 0.19 | 0.65 ± 0.17 | 1.0324722 | 0.56 |
| Right | 0.77 ± 0.17 | 0.68 ± 0.15 | 2.2280953 | 0.07 |
| Rear | 1.11 ± 0.21 | 1.34 ± 0.20 | -5.4464854 | <0.001** |
| Temporal | 0.34 ± 0.32 | 0.60 ± 0.31 | -5.9665963 | <0.001** |

**Table 2**. Transformed Spearman Correlation values for front, center, posterior, and temporal regions, and left and right hemispheres, in four different frequency bands in Two-Object Block. Note: **p* < 0.05, ***p* < 0.01.
